# Daughters Lost!

**Published:** 1867-11

**Authors:** 


### DAUGHTERS LOST!

Thirty thousand daughters are said to earn a precarious living by lives of shame in New-York city alone! Their average lives are four years; seven thousand every year die in sickness, poverty and degradation; yet, there was a time when each one of those daughters was as pure as a dew drop; was cared for so tenderly; was pressed to a fond mother's bosom, how lovingly, no pen can describe, no lip portray! but all were not born in poverty and ignorance; but a few were daughters of the rich manufacturer, of the wealthy merchant, of the proud and haughty banker; but almost all were driven to infamy by one common cause, want; the want sometimes of a hundred dollars, of five, of one!! yes, reader, a dollar would have saved from gnawing hunger, and saved from that, the first crime would not have been committed; and here we come to the practical point, personally applicable to every parent, and which, if heeded, would save ten thousand cherub daughters every year, either from lives of shame, from premature and desperate and ill assorted marriage or incipient steps towards drunkeness or the mad-house. This appeal is made, O how earnestly, to mothers especially; as by a loving and affectionate persistance, they can, in a vast multitude of cases, accomplish the desired object, and then, if called early or suddenly, how peacefully they might die under the delightful assurance, "my daughter can never come to absolute want."

"Don't let poor Nelly starve," was the dying utterance of one of the greatest names in modern history; a name which could have commanded vast sums of money at one time; but no provision was made for "Nelly" in prosperity, and she did die in abject poverty.

There is a large establishment in New-York which never has a vacant room; it is a refuge for the widows and daughters of men who were at one time prominent, influential and rich; but who, in the progress of events, died in poverty. In one respect it is a beautiful sight; to look upon so many old faces, with intelligence, cultivation and refinement depicted in every lineament; themselves so tidy, so cheery, so ladylike, and feel that they can never again come to want. Each one is required to do as much as possible towards furnishing their own rooms, and in addition to do what they or their friends can, in supplying money for food, fuel and other expenses; what they cannot do is made up by the contribution of the large hearted men and women of wealth and position in New-York. But what sad memories must sweep across their hearts at times, in thinking of days long ago! when they lived in wealth, and luxury, and magnificence; and when to have laid by enough to have yielded an annual interest of a small sum, would have been considered too insignificant to have been worth a second thought; and now, to live on the charity of others; without a home, without a shelter, not even a pillow to rest the grey head upon, but comes from pitying hearts, whom they never knew.—If three thousand dollars were laid aside for every wife and as much for each daughter in national securities, without any power to use the principal, what an amazing comfort it would be, to know that eighteen or twenty dollars would be coming in every month with perfect certainty; and that without any other trouble than that of presenting a scrip of paper at the bank; no fruitless dunnings; no risk of angry, or impatient looks, or haughty payment; or of mean dismissal, with its impertinent order, to "call again." Twenty dollars a month! it does not seem much now; a ludicrous sum to many a reader; but a worthy woman came to my door less than forty eight hours ago, and how piteously did she plead for only two dollars; it was Saturday, just at sun down, and if not paid that night, she and all her children would have to sleep on the pavement or in the "lock up." Twenty dollars a month! There is many a neat, tidy, religious family in the country, who would be glad to receive as a member of their household a respectable woman for three dollars a week, regularly and cheerfully paid, to whom three hundred dollars a year, certain, prompt, without any effort, seems a large sum; and the owner of the income would be considered rich and would be treated as such; for let it be remembered, that it is a phase in our human nature, that an indebtedness, always paid promptly, cheerfully, courteously, invests the person who pays it, with an ability to pay any other greater sum, only if the payor will have the gumption to keep wholly to himself, the amount of his income; never giving, in the most remote manner any clue to the length of his purse, being careful as to one point only, to anticipate a claim, even if it be but for an hour; to pay it with some cheerful remark, without waiting to be asked for it. This would bring from ten million shopkeepers and dealers the voluntary and heartfelt "testimonial": She is a lady, every inch of her." "He's a perfect gentleman."—That some such provision would save many a loving wife, many a daughter who this hour moves with queenly steps and grace over the velveted floors of "the Avenue," from the bawdy house, the gutter, the hospital, the penitentiary and the asylum, is demonstrable, cannot be denied.—But why have government securities been given the preference? First and foremost, because it is a perfectly safe investment as long as the government stands, and if it falls, there can be but one other want, "to lay me down and die!"

Second, nobody need then or can easily find out your income, and you can bid defiance to the tax-gatherer, for his book is open to every newspaper in the land.

Third, there is no delay in payment, and that most distressing process of "dunning" is avoided; no listening to piteous tales of debtors; no foreclosing of mortgages; no wearing out of shoe leather; no mental disturbances of any sort; the avoidance of all these makes a government payment worth double the amount, to every high toned sensitive, liberal mind.

Mother, think of this, and this very night, or the first time your husband is in a loving mood, talk it over with him, and give him no rest until the deed is done and the papers irrevocably signed, and then you need not be distressed in a dying hour with the piteous petition "Don't let poor Nelly starve."

